# Helping Health Care Providers and Clinical Scientists Understand Apparently Irrational Policy Decisions

**DOI:** 10.7759/cureus.936

**Published:** 2016-12-21

**Authors:** Sandor J Demeter

**Affiliations:** 1 Diagnostic Imaging, University of Manitoba/WRHA-HSC

**Keywords:** health economics, biomedical ethics, health law, epidemiology, health care policy, health care professionals, public health, health policy

## Abstract

Health care providers (HCP) and clinical scientists (CS) are generally most comfortable using evidence-based rational decision-making models. They become very frustrated when policymakers make decisions that, on the surface, seem irrational and unreasonable. However, such decisions usually make sense when analysed properly. The goal of this paper to provide a basic theoretical understanding of major policy models, to illustrate which models are most prevalent in publicly funded health care systems, and to propose a policy analysis framework to better understand the elements that drive policy decision-making. The proposed policy framework will also assist HCP and CS achieve greater success with their own proposals.

## Introduction and background

The Oxford dictionary defines policy as "A course or principle of action adopted or proposed by an organization or individual" [1]. It is difficult to adopt a policy without resources so I would expand this definition to the allocation of resources by those who have the authority to do so and policy analysis explores factors that drive such decisions. Classical policy models include incremental, rational, or mixed incremental/rational. The Canadian Health Care system will be used as an example of a publicly funded health care system.

Incremental models were defined in 1959 by Lindblom who referred to them as the science of “muddling through” [2]. Incremental models involve a sequence of disconnected small steps, or as Lindblom describes, “successive limited comparisons”, which, in aggregate, do not necessarily have any meaning or general direction. Lindblom would suggest that the majority of Canadian public policy fits the incremental model.

Rational policy models are grounded in some well-defined economic, scientific, social, or other framework with a pre-hoc defined ultimate goal [3]. The current Canadian Government has espoused an “evidence-based” policy approach [4], which, on the face of it, is in keeping with a rational policy model. Setting and working towards identified carbon release targets to address global warming would be an example of a rational policy approach.

Policy decisions in the real world can be complex and may contain both incremental and rational components. Etzioni described this middle ground as a hybrid “mixed-scanning” model, which involves making small incremental decisions leading to some overall rational goal [5].

**Figure 1:**
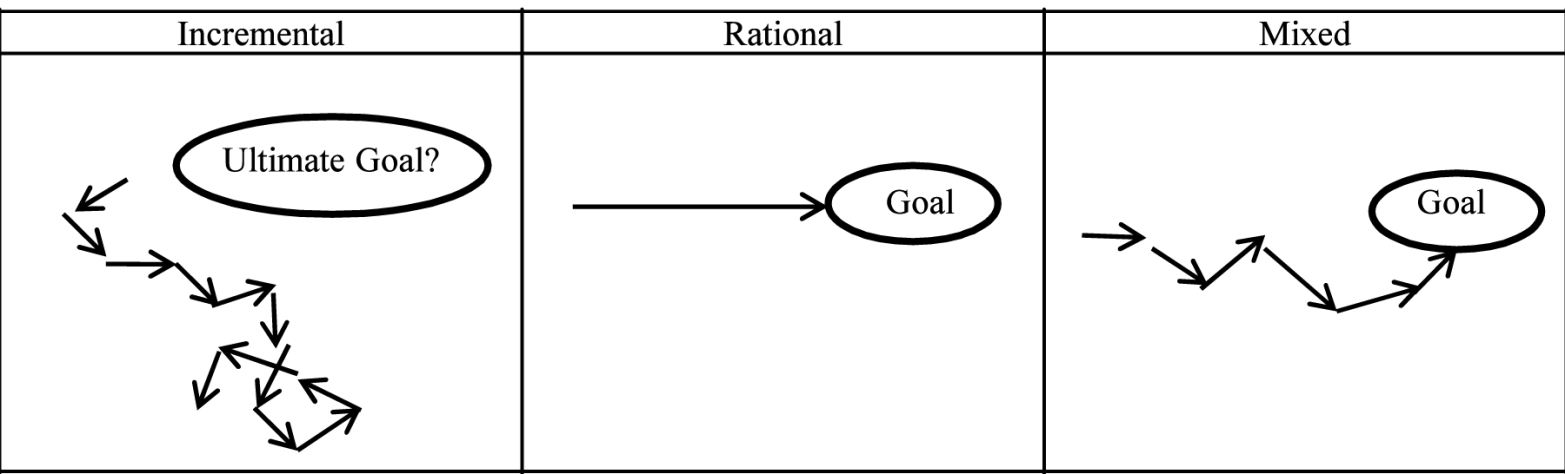
Comparison of Incremental, Rational, and Mixed Policy Models For the three policy models illustrated, each arrow represents a decision as a vector. In the incremental model, the overall direction of the arrows is not well defined, whereas for the rational and mixed models, there is pre-determined ultimate direction corresponding to a stated goal.

Figure 1 contrasts these three models. Each arrow defines a separate decision which either does (i.e., rational and mixed) or does not (i.e., incremental) lead to an overall goal.

In an era of evidence-based health care, HCP and CS strive to work in a scientific rational policy arena. However, it is naïve to think that policy decisions are only driven by scientific reasoning. Policymakers are influenced by many other factors, in addition to what makes sense scientifically.

**Figure 2:**
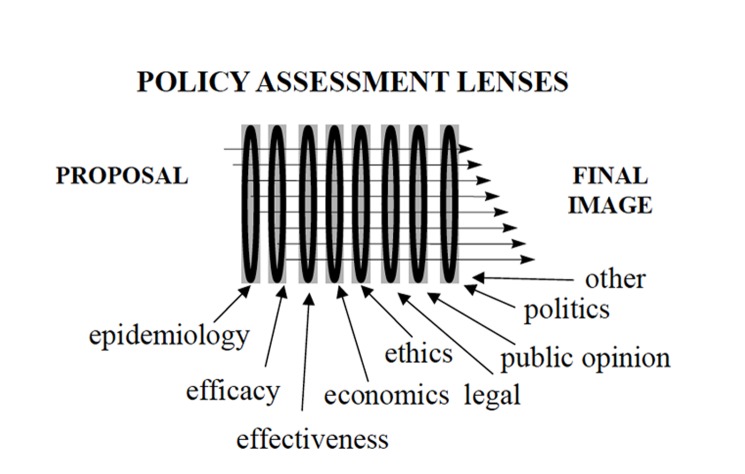
Framework for Policy Assessment and Analysis The figure illustrates that policy proposals are shaped and focused by various factors represented by “policy assessment lenses”.  Understanding how the proposal will be shaped by the various lenses, resulting in an ultimate “final image”, will help predict the ultimate success or failure of the proposal.

Figure 2 outlines a series of “policy assessment lenses” which a policymaker may use to shape and review a proposal leading to the final decision. These lenses could also be considered as a set of potentially competing constraints that a policy maker has to consider and reconcile. The goal of the “proposer” is to sufficiently understand these “policy assessment lenses”, such that their proposal results in a favourable final image after it has gone through the applicable set of lenses. In other words, if the proposer sufficiently understands the mindset of those ultimately making the decision, they can anticipate and take into account all relevant considerations to ensure that the final image, which drives the policy decision, is as expected.

The following is a brief synopsis of the individual policy assessment lenses. HCP and CS are generally most experienced, and comfortable, with the first four “lenses”, i.e. epidemiology, efficacy, effectiveness, and ethics.

## Review

### Epidemiology

Epidemiology is the study of the occurrence and distribution of health-related states or events in specified populations, including the study of the determinants that influence such states, and the application of this knowledge to control the health problems [6].

From a policy perspective, the greater the “burden” of disease or illness (i.e., the number of individuals affected), the more likely the issue will be recognized as being important. In addition, specific target populations may also influence recognition. Examples include children, women (e.g., breast cancer), men (e.g., prostate cancer), and the elderly (e.g., dementia). There are also issues that cut across target populations, such as initiatives focused on drunk driving (e.g., Mothers Against Drunk Driving - MADD).

### Efficacy

Efficacy relates to how well the proposal performs under ideal controlled conditions, with multicentre randomized controlled trials being the most representative methodology of such. In other words, what are the best outcomes that can be expected in an ideal setting?

### Effectiveness

Effectiveness relates to how well the proposal performs under real world conditions, with prospective observational and retrospective studies being the most representative methodologies of such.

### Economics

Economics is an important consideration in publicly funded health care systems. This is especially evident in pharmacare where governments have to make funding decisions across a field of competing pharmacological interests. In the public realm, resources are scarce and getting the most net benefit amongst a number of competing options is the goal.

If a new initiative is less expensive and more effective than the current standard of care, then it “dominates” other strategies. In contrast, if a new initiative is more expensive but less effective, it is unlikely to be adopted. However, if a new initiative is more expensive and more effective, how much “more effective” does it need to be to justify adoption?  This question is addressed by four health economic indicators: cost minimization (CM), cost-effectiveness (CE), cost utility (CU), and cost-benefit (CB).

In CM, the outcomes of the options being considered are equal and the most economical option becomes the most desirable.

CE and CU are measures of the net additional benefit gained by the new proposal compared to the existing standard(s) of practice. CE is the net cost ($) per additional natural units, e.g., year of life gained. CU is the net cost ($) per additional quality-adjusted units, e.g., year of life gained (e.g., QALY). CE and CU are commonly expressed as incremental cost-effectiveness ratios (ICER). The question of what is an acceptable threshold, or incremental cost-effectiveness ratio (ICER), is debatable. In 1992, Laupacis, et al. [7] proposed that, if the ICER is below $20,000 per additional year of life saved, the proposal is desirable, between $20,000 to $100,000, it could be considered, and above $100,000, the proposal may not be considered cost-effective. The UK National Institute for Health and Clinical Excellence (NICE) [8-9] has proposed an ICER ratio threshold of between £20,000 to £30,000 (~$38,000 to $57,000 CAD) [10].

One of the limitations of the CE/CU approach is that it is difficult to compare health policy proposals with different outcomes. For example, when comparing quality adjusted life years (e.g., chemotherapy) against cavity-free children (e.g., a pediatric cavity prevention program), the CE/CU approach will not work. Policymakers, especially at higher levels, may want to compare and contrast long lists of possible health care resource allocations. This is where a CB economic analysis can be applied. In a CB analysis, all outcomes, such as potential years of life saved, disability-free days, progression-free survival, etc., are converted to dollar values. Putting a value on a year of life is somewhat controversial but it is not dissimilar to setting a CE/CU threshold. The dollar values for the various outcomes are generally estimated through a “willingness to pay” exercise where individuals are asked how much they would be willing to pay per unit of outcome. This allows a rank listing of diverse health programs and services comparing how much each item costs compared to the dollar value of the outcome. CB is commonly referred to as getting the best “bang for your buck”.

Table 1 summarizes the basic characteristics and differences between CM, CE, CU, and CB.

**Table 1:** Types of Health Economic Analysis Economic studies have two components: cost (always $) and outcomes (natural units, quality adjusted units or $). Various combinations of these two components result in four distinct types of economic analyses, which are listed in the first column. QA = quality adjusted, QALY= quality adjusted life years

	Cost	Outcome	Incremental Difference
Cost Minimization	$	=	Lowest cost
Cost Effectiveness	$	Natural units	$/additional year of life
Cost Utility	$	QA units	$/additional QALY
Cost Benefit	$	$	$/$

### Ethics

In health care, the most commonly cited ethics framework has been published by Tom Beauchamp and James Childress [11].

The four key elements of their framework include:

Autonomy – patients have a right to choose or refuse care Beneficence – health care workers have a duty to act in the best interest of their patients Non-maleficence – “first do no harm” (primum non nocere). Justice – relates to the fair and equitable distribution and access to health care resources

Ethical issues can be complex, especially when there is a conflict between ethical elements. For example, a patient may want a health care service for which the health care provider feels may cause them more harm than good, resulting in a conflict between patient autonomy and non-maleficence.

The current debate about low-dose CT screening for lung cancer is a good example of the challenges of making programmatic and resource decisions at a targeted population level. Does low-dose CT lung cancer screening cause more good than harm? Initial results from the National Lung Screening Trial (NLST) are promising from an epidemiology point of view [12]. However, the very low overall positive predictive value for positive screening rates of only 3.8% have raised concerns about the potential harm of false positives. Croswell, et al. commented, “Risks for false-positive results on lung cancer screening tests are substantial after only two annual examinations, particularly for low-dose CT. Further study of resulting economic, psychosocial, and physical burdens of these methods is warranted.” [13].

### Legal

Policy decisions are strongly influenced by existing legislation. If decisions are contrary to such, they can be challenged. Governments may choose to amend legislation to accommodate new policy directions but this takes time, especially if the change is beyond an order in council (e.g., commonly used to change regulations). For example, a substantive change to an act generally requires three readings in the house (legislature at the provincial level and parliament at the federal level), approval of the senate for federal statutes, and royal ascent at both levels.

As well, existing laws can be challenged. For example, the Canadian Supreme Court recently ruled (February 2015) that laws prohibiting assisted death of terminally-ill patients were unconstitutional and violated Section 7 of the Canadian Charter of Rights and Freedoms (i.e., the right to life, liberty, and security of the person [14]). The Supreme Court gave the Canadian government one year, with a subsequent extension, to make changes to Canadian Criminal Code to allow for the provision of physician-assisted death [15-16]. Amendments to Canadian Criminal Code allowing such received Royal Assent on June 17, 2016. However, the amendments are not without controversy as they apply to those for whom death is “reasonably foreseeable” but not necessarily for those with chronic, insufferable conditions for whom death is not “reasonably foreseeable”. Some feel the amendments are too restrictive, given the original direction of the Supreme Court.

In addition, health care policy may be influenced by multiple levels and branches of government (e.g., Federal, Provincial, Municipal, etc.) and this needs to be taken into account when analysing the feasibility of the proposal.

### Public opinion

Elected officials constitute a good portion of those who directly or indirectly influence law-making and policy decisions. The opinions of the electorate, and the public, at large, can be influential, especially when combined with election cycles.

For example, Sznitman, et al. [17] have published on how public opinion on the medical effects of cannabis may influence public policy relative to legalizing medical cannabis use.

Burstein [18] has conducted a relatively comprehensive review of the impact of public opinion on public policy. He concludes that, while public opinion generally does have an impact on policy, the relative influence of relevant factors, such as issue salience, source of opinion (e.g., interest group, political party, elite, or general public), secular trends, and the ability to generalize the issue, are not well known and have not been well quantified in the literature.

### Politics

Politicians, collectively referred to as “government”, are responsible for making laws and having a significant influence on resource allocations. At the end of the day, and put simplistically, the government can only influence behaviour in three ways: incentives (e.g., tax breaks, rebates, transfer payments, direct payment to individuals, etc.), disincentives (e.g., criminal, civil, or bylaws), or exhortation (e.g., promises). In the Canadian system, as is the case in most democratic countries, politicians are elected for fixed terms. As such, politicians, and especially the political party that has a majority or minority lead in Parliament or Legislatures, are sensitive to election cycles and the need for appropriate “positioning” for re-election purposes. This may disadvantage longer term projects that extend beyond election cycles.

A proposal, which takes into account election cycle timing and provides positive feedback for a politician or a political party, will probably fare much better than one that is naïve to such factors.

In addition, understanding, in real time, what is driving public opinion is important as resources may be directed accordingly. One may ask how does their proposal relate to or address issues important to the government. The more aligned the proposal is to the mandate or issue of the day, the more likely it will be supported.

Political ideology is also important. For example, if a ruling government believes that publicly funded services should be delivered in publically administered facilities, it may be difficult to gain approval for a publically funded service being delivered in a private setting.

### Other

There are a number of other factors which may influence policy and resource allocation. They include security crises (e.g., airline security measures post-major incidents), natural disasters (e.g., fires, floods, tornados, and earthquakes), epidemics (e.g., meningitis, influenza, Zika virus), social/economic factors (e.g., societal unrest or economic downturns), and international affairs (e.g., refugee crises, natural disasters, war), to name a few.

### Case study - MRI wait times

MRI services were first offered Winnipeg at Saint Boniface General Hospital in 1990 [19]. There are currently 10 MRI units in Manitoba (MB) of which eight are in the Winnipeg Regional Health Authority (WRHA) [20]. 

**Figure 3:**
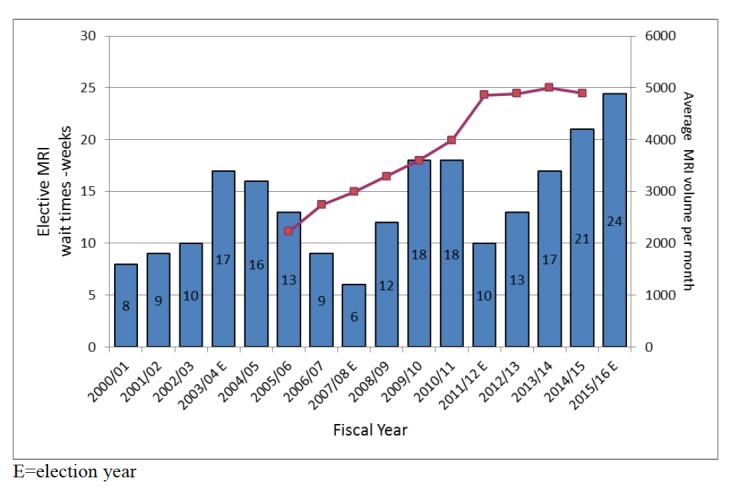
MRI Wait Times and Volumes by Time Temporal elective MRI wait times (weeks) [blue bars] and monthly MRI volumes [red line] are presented between the years 2000 to 2016 for wait times and 2005 to 2016 for volumes. Non-sustained nadirs in elective MRI wait times are observed corresponding to the 2007/08 and 2011/12 election years but not for the 2003/04 and 2015/16 election years.

Figure 3 illustrates trends in average elective MRI wait times, in weeks, between the fiscal year 2000/2001 to 2015/2016 and corresponding average monthly MRI volumes between 2005/2006 to 2015/2016 for the WRHA. Data from monthly “WRHA Diagnostic Imaging Program Waiting List Status Reports” are submitted to Manitoba Health on a monthly basis. These data are then summarized and published on the internet as Manitoba Health “Wait Time Reports” [21]. The “E” after the fiscal year indicates an election year. The New Democratic Party formed a majority provincial government for the first three indicated elections and the Progressive Conservative Party won a majority mandate in the 20015/16 election. For the 2007/08 and 2011/12 election years, there is an associated, relatively short-lived, significant decline in MRI wait times (blue bars). However, this decline is not observed for the most recent 2015/16-election year. It is also noted that the average MRI volume (red line) increased steadily until 2011/12, where it rose more steeply until plateauing starting in 2012/13.

This data suggests that factors, other than clinical demand, appear to drive health care resource allocation. Increased capacity during the 2007/08 and 2011/12 election years was achieved by increasing shifts (i.e., adding evening and night shifts) as well as by the temporary addition of a portable MRI for the 2007/08 election year. It would appear that factors, such as election cycles and associated or anticipated public opinion, drove such resource allocation decisions. That is, increased resources were allocated to reduce the elective MRI wait times to correspond to Provincial election cycles. It is interesting to note that over three election cycles, this was not observed in the more historic 2003/04, and more recent April 2016, MB Provincial election.

## Conclusions

On the face of it, policy decisions can appear irrational. However, when a more comprehensive set of factors is considered, such decisions may be made understandable. It is important to be comfortable with factors outside of scientific reasoning, such as public opinion, politics, economics, and legal issues. This will achieve two goals: first, a better understanding and comfort level with what influences policy decisions, and second, how to make one’s own research or health service proposals more relevant and successful. It is hoped that the provided model of a set of policy assessment “lenses” will help achieve these goals.
